# Supplementary material for: Alternative splicing controls teneurin-3 compact dimer formation for neuronal recognition
Source: Nat Commun. 2024 Apr 29;15:3648. doi: 10.1038/s41467-024-47763-x (PMC11058771; doi:10.1038/s41467-024-47763-x)
Supplement: Supplementary file 1 — Supplementary Information [file 41467_2024_47763_MOESM1_ESM.pdf]

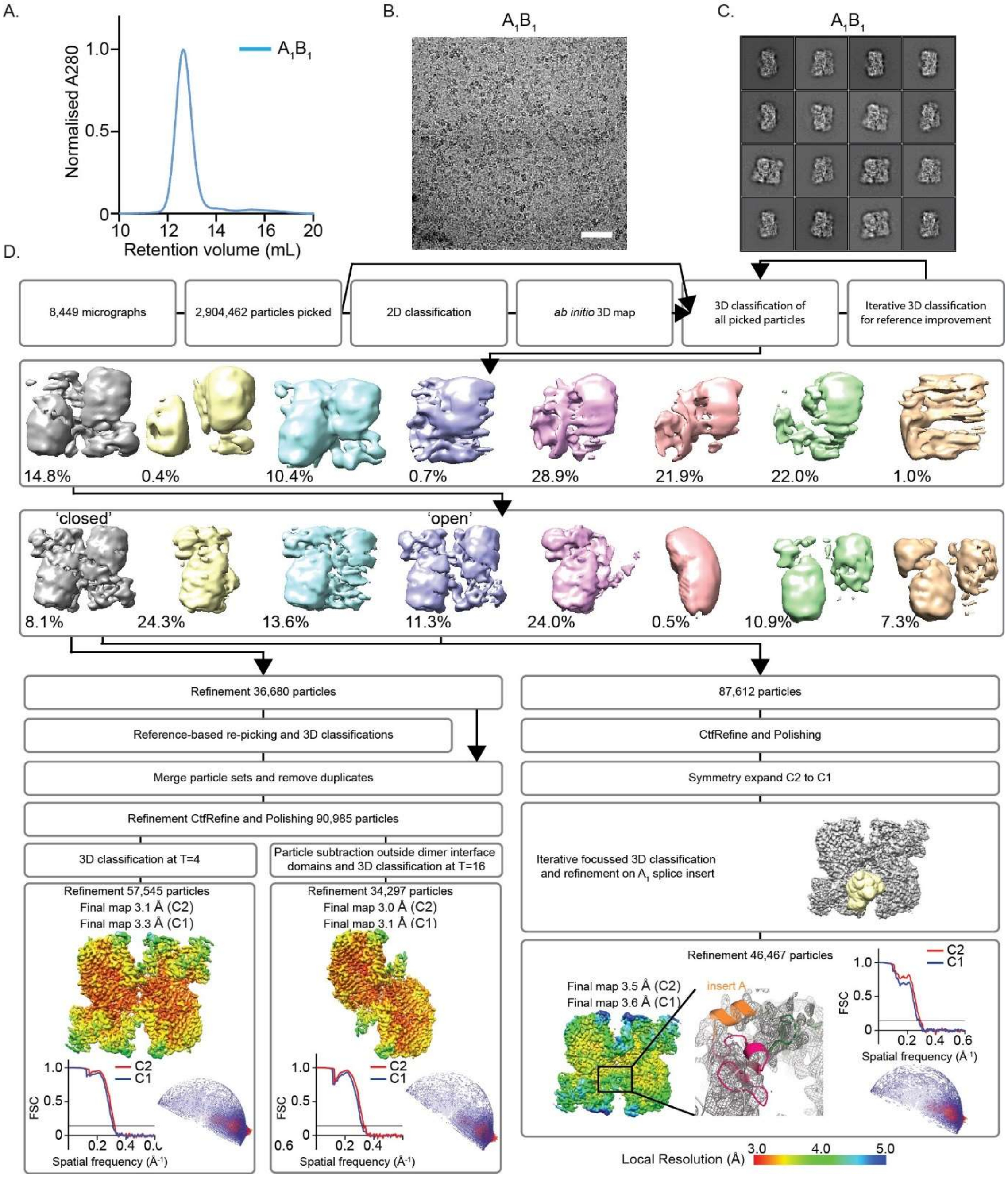

Supplementary Figure 1: Purification, cryo-EM data collection and reconstruction of teneurin-3 A_1B_1 compact dimer. **A)** Size exclusion chromatography (SEC) trace of the Ten3-A_1B_1 ectodomain after nickel affinity purification. **B)** Representative cryo-EM micrograph of vitrified Ten3-A_1B_1 ectodomain. Scale bar is 50 nm. **C)** Single-particle analysis (SPA) 2D classes of compact dimeric Ten3-A_1B_1 particles in 'closed' conformation. **D)** SPA workflow for the reconstruction of cryo-EM electron density of compact dimeric Ten3-A_1B_1. Left: reconstruction workflow of the full dimeric map. Second to left: dimeric map reconstruction after particles subtraction around interface domain. Third to left: C2-to-C1 symmetry-expanded reconstruction of subunit with density outside YD through Tox-GHH subtracted. Right: focussed classification and refinement of splice insert A (orange helix) with dimers from the closed conformation and more flexible open conformation. Corresponding local resolution values, Fourier shell correlation (FSC) graphs, and angular distribution of particles in final refinement are shown for each map. FSC cut-off is 0.143. Source data for panels A and D are provided as a Source Data file.

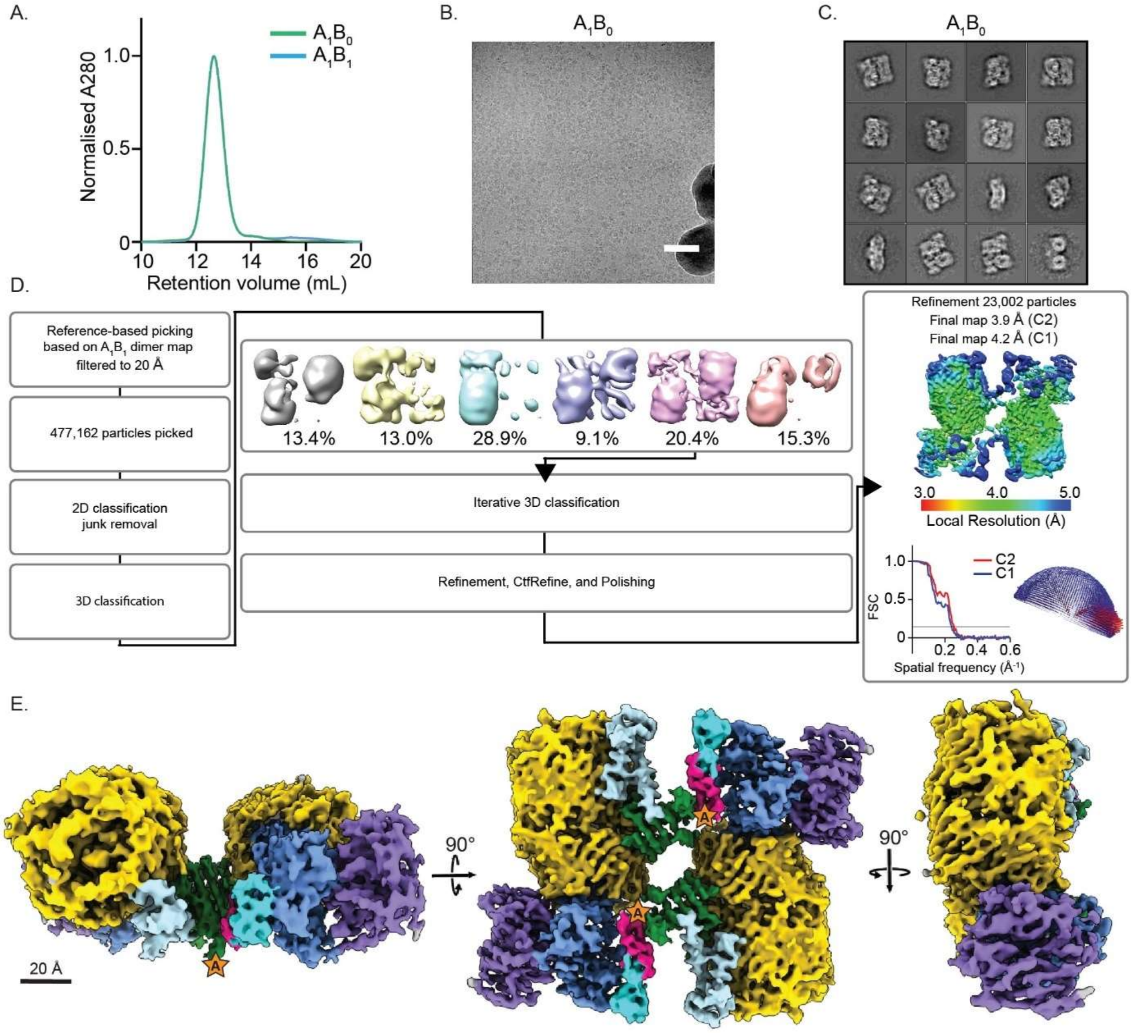

Supplementary Figure 2: Purification, cryo-EM data collection and reconstruction of teneurin-3 A_1B_0 compact dimer. **A)** Size exclusion chromatography (SEC) trace of the Ten3-A_1B_0 versus A_1B_1 ectodomains after nickel affinity purification. Traces essentially overlap. The A_1B_1 trace is equal to that in Supplementary Fig. 1A. **B)** Representative cryo-EM micrograph of vitrified Ten3-A_1B_0 ectodomain. Scale bar is 50 nm. **C)** Single-particle analysis (SPA) 2D classes of compact dimeric Ten3-A_1B_0 particles in 'open' conformation (no YD-YD contact). **D)** SPA workflow for the reconstruction of the cryo-EM density map of compact dimeric Ten3-A_1B_0. Corresponding local resolution values, Fourier shell correlation (FSC) graphs, and angular distribution of particles in final refinement are shown. FSC cut-off is 0.143. **E)** Three different views of the density map of the teneurin-3 A_1B_0 compact dimer at 3.9 Å resolution (EMD-19409 [https://www.ebi.ac.uk/emdb/EMD-19409]). Domains are coloured corresponding to the linear representation in Fig. 1A. Positions of the splice insert A are indicated with stars. Scale bar is 20 Å. Source data for panels A and D are provided as a Source Data file.

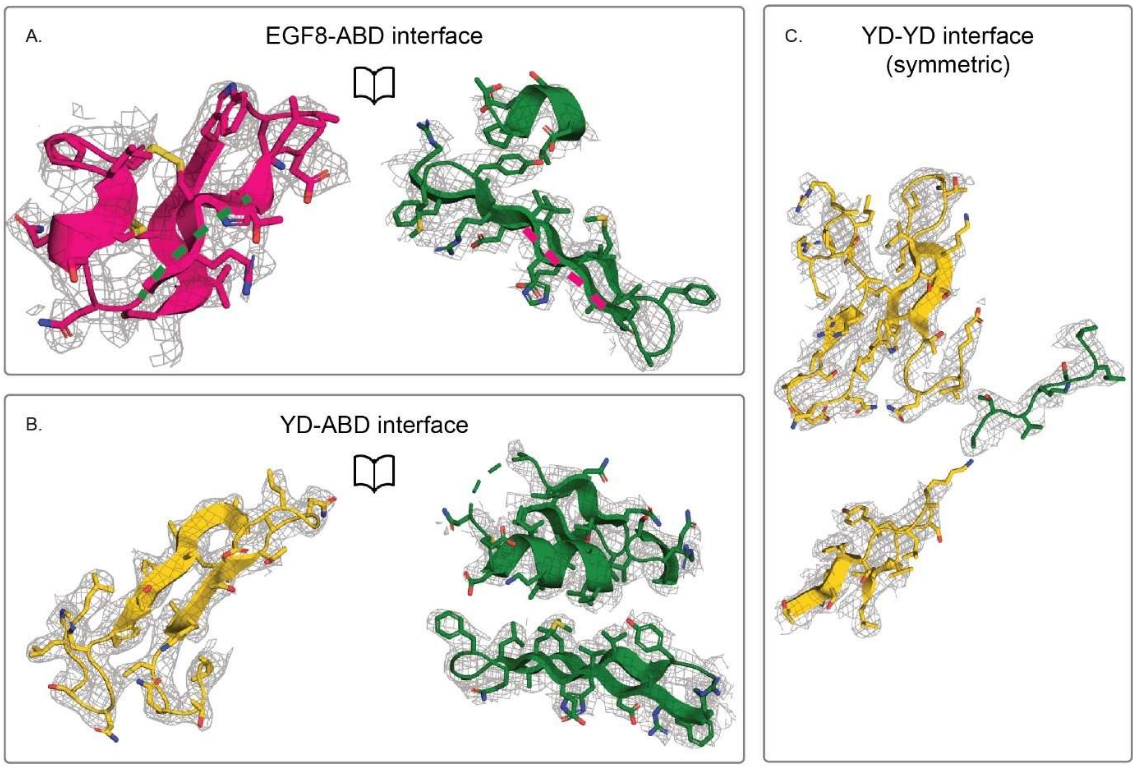

Supplementary Figure 3: Cryo-EM densities for secondary structure elements in the teneurin-3 compact homodimeric interface of the A_1 isoforms. **A)** Open book representation of the secondary structure elements within the EGF8-ABD interface I. Dashed lines in the colour of the complementary domain indicate the extended β-sheet contact. **B)** Open book representation of the secondary structure elements within the YD-ABD interface II. **C)** Secondary structure elements within the YD-YD interface III on the side of one non-compact subunit. All density is visualised at a contour level of 4.2 RMSD. Domains are coloured according to colouring in Fig. 1.

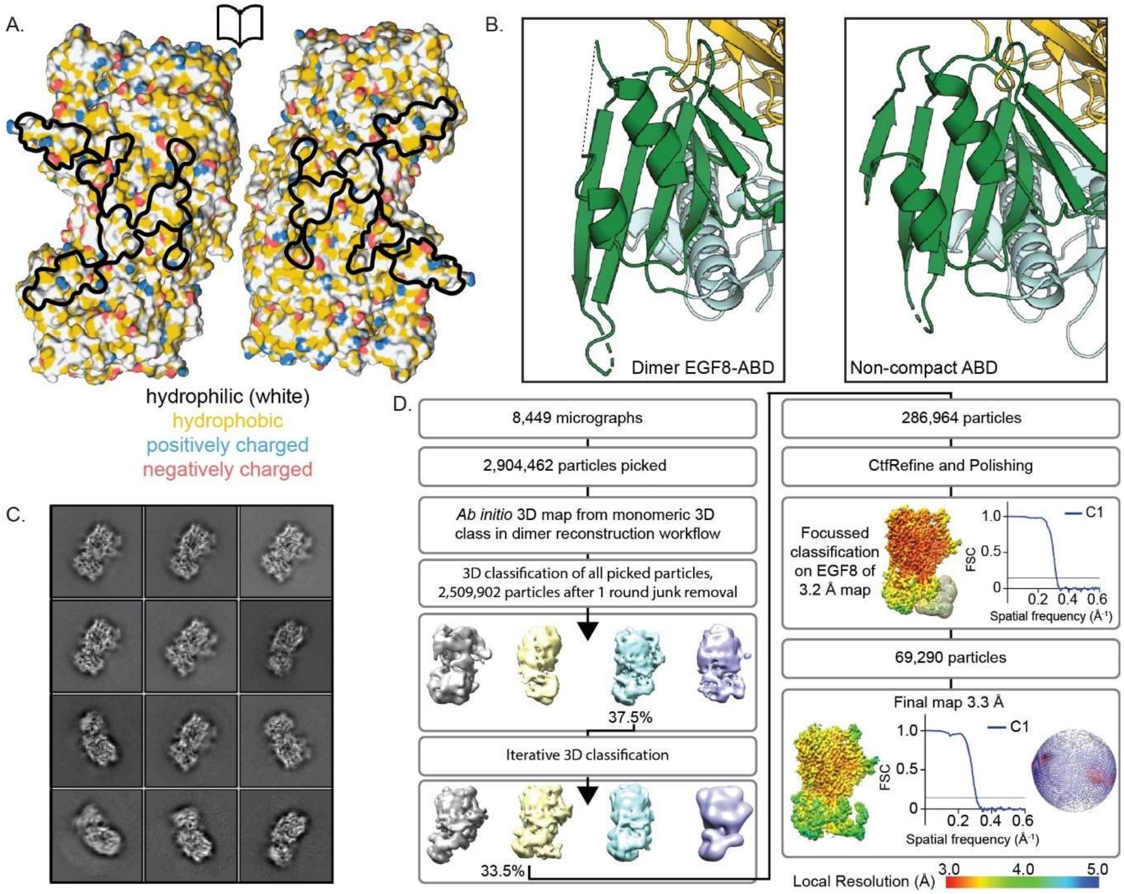

Supplementary Figure 4: Teneurin-3 A_1B_1 compact interface analysis and non-compact subunit reconstruction. **A)** Open book electrostatics and hydrophobicity surface representation of the Ten3 compact dimer. Black lines indicate the outline of compact dimer interface. **B)** Structural comparison of the Ten3-A_1B_1 non-compact subunit (PDB:8R51 [https://doi.org/10.2210/pdb8R51/pdb]) ABD versus the compact dimeric (PDB:8R50 [https://doi.org/10.2210/pdb8R50/pdb]) EGF8-contacting ABD. The non-compact ABD displays the presence of an additional β-strand at solvent-facing edge of the ABD β-sheet. Missing residues in the non-compact subunit are indicated with dashed black line. **C)** Single-particle analysis (SPA) 2D classes of Ten3-A_1B_1 non-compact subunit particles after focussed classification on EGF8. **D)** SPA workflow for the reconstruction of the Ten3-A_1B_1 non-compact subunit, including subsequent focussed classification and refinement of the EGF8-containing particles. Corresponding local resolution values, Fourier shell correlation (FSC) graphs, and angular distribution of particles in final refinement are shown for each map. FSC cut-off is 0.143. Source data for panel D are provided as a Source Data file.

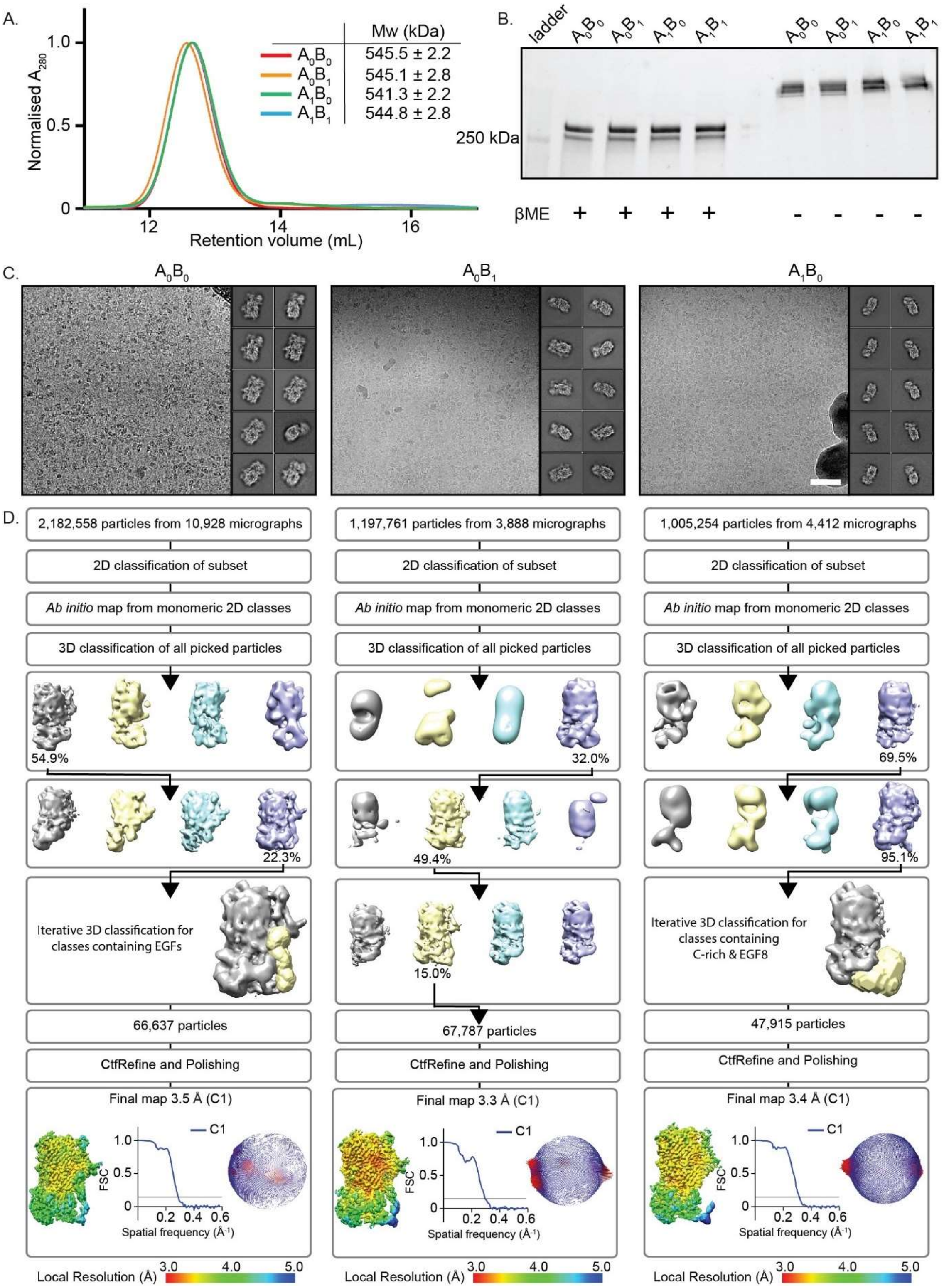

Supplementary Figure 5: Purification, cryo-EM data collection and reconstruction of teneurin-3 A_0B_0, A_0B_1, A_1B_0, non-compact subunits. **A)** Size exclusion chromatography (SEC) trace of all Ten3 ectodomains after nickel affinity purification. Traces essentially overlap. Inset displays the weight-averaged molar mass determined from multi-angle light scattering (MALS) data (mean ± SEM) of all Ten3 isoform at a protein concentration of 1.0, 0.5, and

0.25 mg/mL in presence of 2 mM Ca^{2+} directly after exiting the SEC column. The A_1B_0 and A_1B_1 traces are equal to those in Supplementary Fig. 2A. **B)** PAGE gel of all purified Ten3 isoform ectodomain in the presence and absence of reducing agent β-mercaptoethanol (βME). This gel was repeated twice with similar results. Full uncropped image of the gel is available. **C)** Representative cryo-EM micrographs of vitrified Ten3 ectodomain and single-particle analysis (SPA) 2D classes for the non-compact A_0B_0, A_0B_1, and A_1B_0 isoform subunits. Scale bars are 50 nm. Per isoform 2D classes of the final reconstructions from single-particle (SPA) analysis in D are shown. The representative micrograph for the A1B0 isoform is identical to Supplementary Fig. 2B. **D)** SPA workflow for the reconstruction of the cryo-EM density map of Ten3 isoform subunits. Workflows include subsequent focussed classification and refinement of the EGF-containing particles. Corresponding local resolution values, Fourier shell correlation (FSC) graphs, and angular distribution of particles in final refinement are shown for each map. FSC cut-offs are 0.143. Source data for panels A and D are provided as a Source Data file.

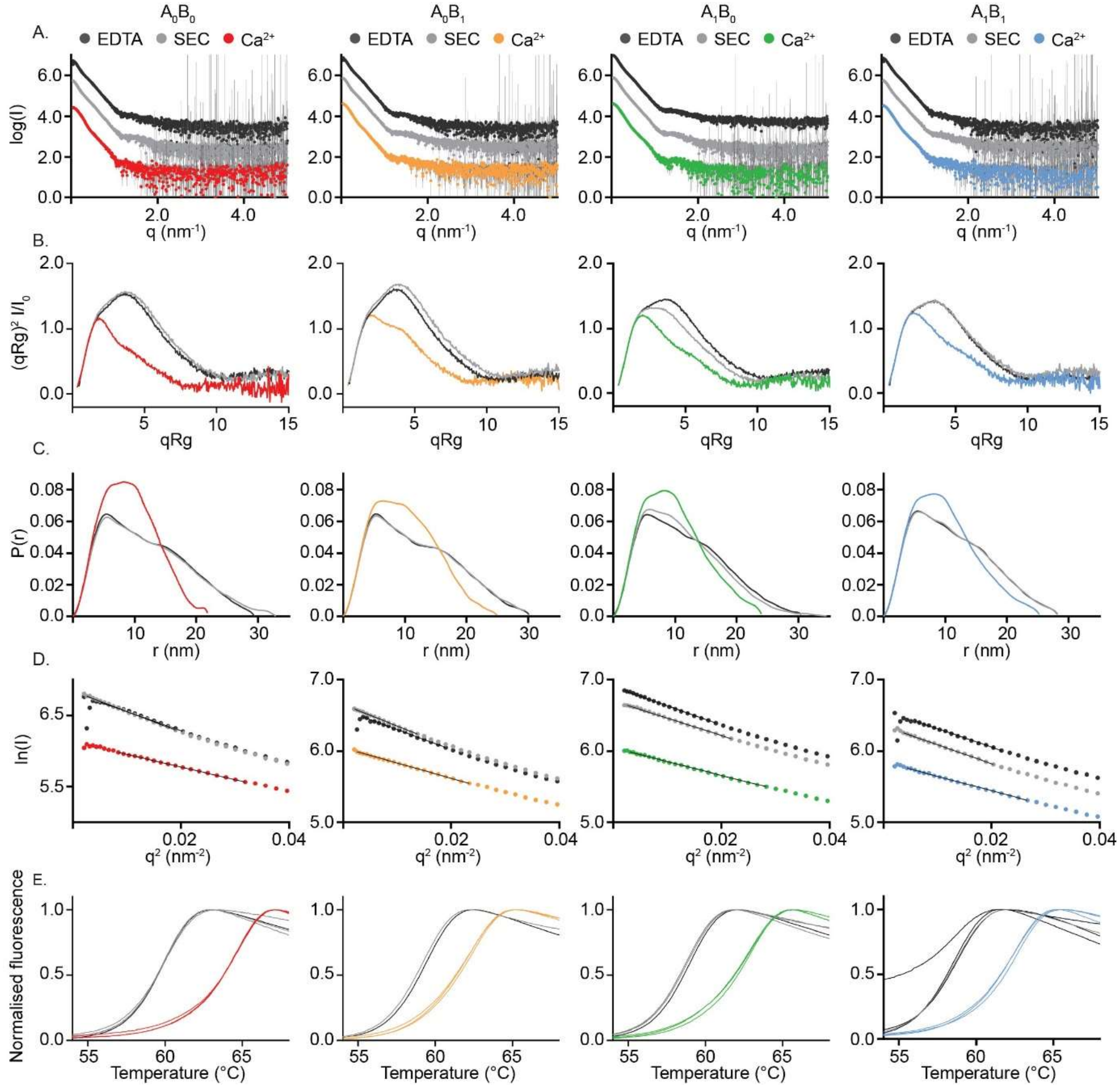

Supplementary Figure 6: The compactness of all four Ten3 isoforms is influenced by the presence of calcium ions. **A)** Small-angle x-ray scattering profile for all purified teneurin-3 (Ten3) isoforms. Scattering curves (left) are shifted by an arbitrary offset along the y-axis for clarity. **B)** Dimensionless Kratky plot for all purified Ten3 isoforms. **C)** Pair-distance distribution, normalized to the area under the distribution, for all purified Ten3 isoforms. **D)** Guinier plot for all purified Ten3 isoforms. Only datapoints in the q^2 range indicated by the Guinier model linear fit (black line) were included in the linear fit. All R^2 values for linear fits are greater than 0.997 (see Table 2), supporting the SAXS data quality. **E)** Melting curves for all purified Ten3 isoforms. Each graph compares the conditions for a Ten3 isoform in the presence (Ca^{2+}) and absence (SEC) of 2 mM calcium, as well as under addition of 5 mM EDTA (EDTA). Source data are provided as a Source Data file.

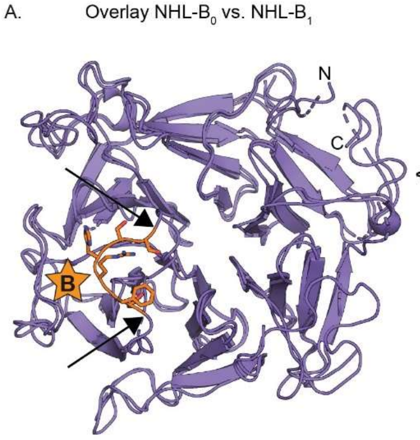

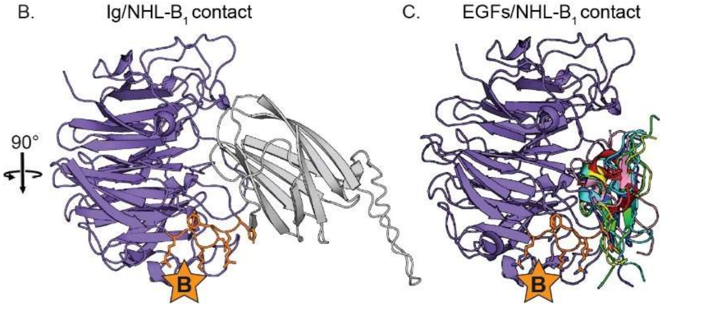

D.

Contacts predicted out of 5 predictions

	Ig	EGF1	EGF2	EGF3	EGF4	EGF5	EGF6
**NHL-B_0**	5/5	5/5	5/5	5/5	5/5	4/5	4/5
**NHL-B_1**	5/5	5/5	5/5	4/5	5/5	4/5	4/5

Supplementary Figure 7: Effect of splice insert B on local NHL domain structure and on interactions with EGF and Ig domains. **A)** Structural comparison of NHL domains lacking (B_0) and containing (B_1) splice insert B. B_0 and B_1 models were built into Ten3-A_0B_0 non-compact subunit and A_1B_1 compact dimer subunit densities, respectively. Splice insert B is shown as orange stick representation. Boundaries of splice insert B are indicated with arrows. N- (N) and C-termini (C) of the NHL domain are indicated. **B)** Colabfold predictions of the NHL-B_1 contact with Ig domain (grey). Splice insert B is shown as orange stick representation, and indicated with a star. **C)** Colabfold predictions of the NHL-B_1 contact with EGF1 through EGF6. Different EGFs predictions have different rainbow colouring, and splice insert B is shown as orange stick representation, and indicated with a star. **D)** Table showing the proportion of predictions containing a direct contact between the EGF or Ig domains and the NHL loop harbouring splice site B - out of 5 total predictions per NHL-EGF or NHL-Ig pair.

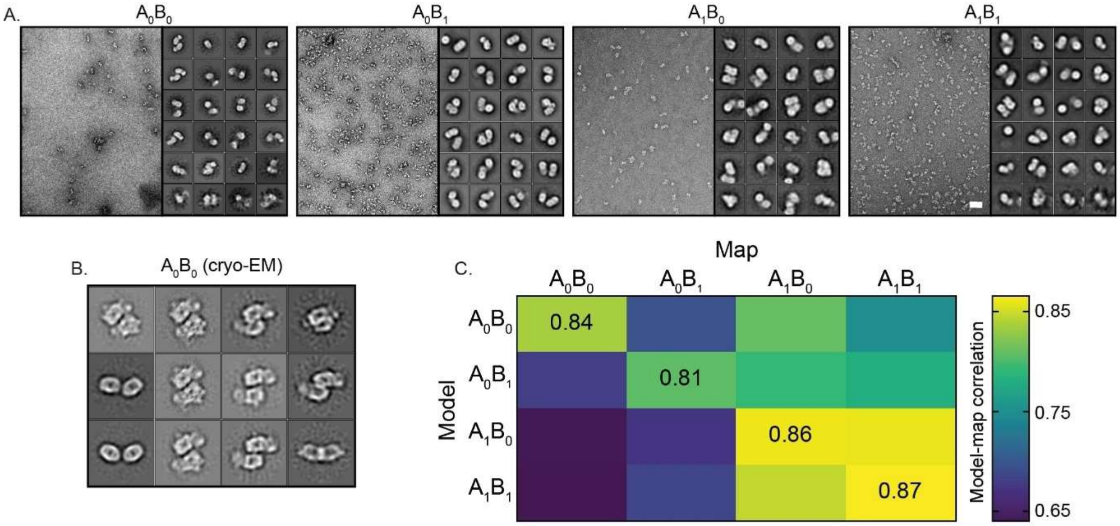

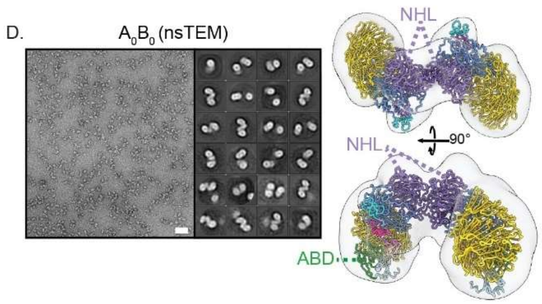

E.

SAXS modelling constraints		χ² of models fit to SAXS data			
Dimer interactions		Ten3-A_0B_0 + Ca^{2+}	Ten3-A_0B_1 + Ca^{2+}	Ten3-A_1B_0 + Ca^{2+}	Ten3-A_1B_1 + Ca^{2+}
EGFs	Superfolds				
2-2 / 5-5	YD-YD	1.33	16.17	5.32	5.73
2-2 / 5-5	FN-FN	3.03	20.49	7.22	7.25
2-2 / 5-5	EGF8-ABD	2.05	10.83	2.68	3.12

Supplementary Figure 8: Teneurin-3 isoform-specific compactness and assessment using negative stain electron microscopy. **A)** Representative negative stain EM micrographs of Ten3 ectodomain and single-particle analysis (SPA) 2D classes of all picked particles for each isoform indicated above. Scale bars is 50 nm. **B)** Cryo-EM SPA 2D classes of Ten3 A_0B_0 in a YD-YD-connected compact conformation. **C)** Quantification of model-to-map fitting in panel D by correlation of the model-derived calculated low-resolution filtered (20-Å) maps with the experimental nsTEM density maps. Heat map representing correlation values for each model to each map, and numbers (on diagonal) in the heat map indicate the highest correlation values per experimental map. Source data for panel C are provided as a Source Data file. **D)** Negative stain TEM micrograph with SPA 2D classes and 3D refinement of the A0B0 NHL-NHL connected compact dimer. Scale bars is 50 nm. **E)** Fits of rigid-body models to SAXS data. Rigid-body models were calculated by restraining the superfold interfaces described in Fig. 5E and the covalent disulfide bonds in the EGF stalk and using the SAXS data corresponding to the isoform for which the superfold interfaces are found, i.e. Ten3-A_0B_0 for YD-YD, Ten3-A_0B_1 for FN-FN and Ten3-A_1B_1 for EGF8-ABD. χ^2 values are calculated by comparing the calculated models against the SAXS data of all isoforms.

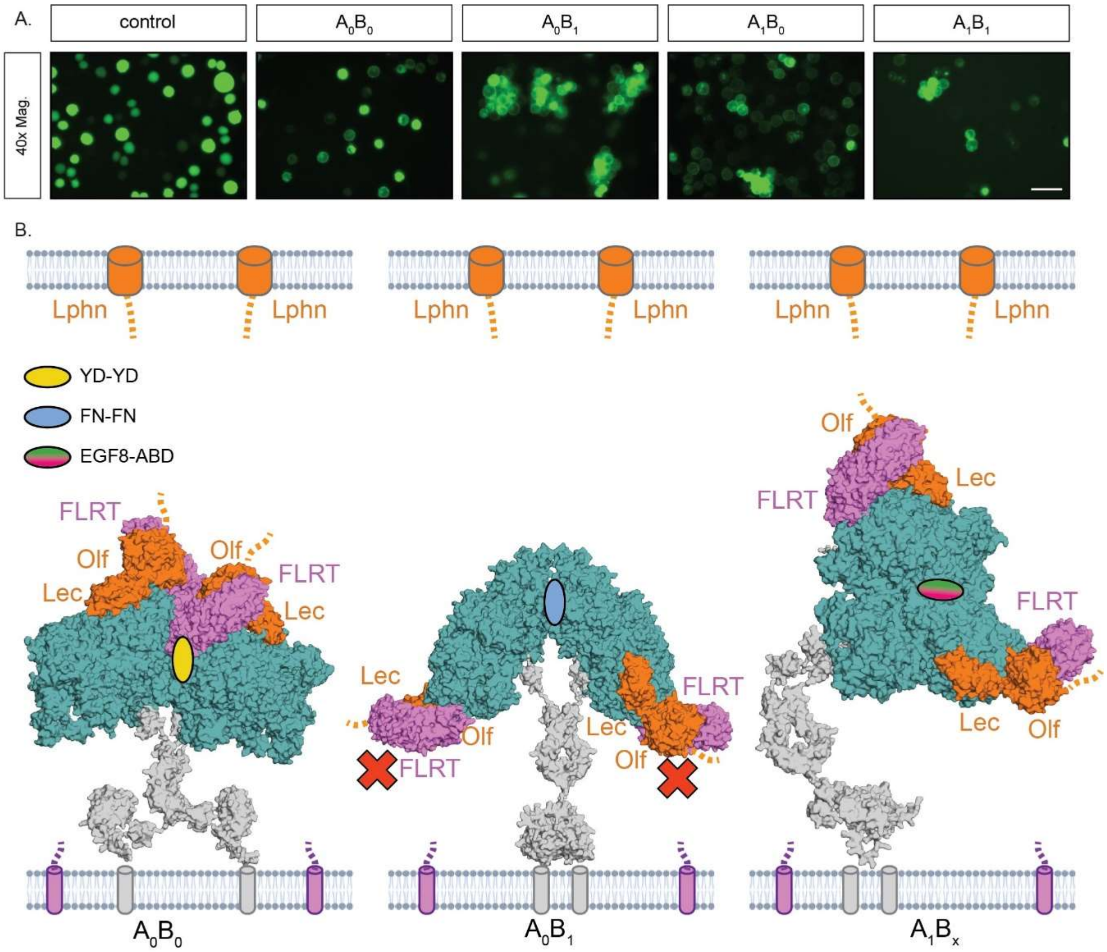

Supplementary Figure 9: Model for isoform-specific teneurin-latrophilin *trans*-cellular complex formation. **A)** Clustering assay of K562 hematopoietic cells electroporated with the mouse Ten3 isoforms at 40x magnification. For all isoforms, GFP signal localises to the membrane in contrast to the soluble GFP that occupies the entire cell area. Scale bar is 100 µm. **B)** Exposure of known teneurin-latrophilin (Olf-Lec, PDB:6SKA [https://doi.org/10.2210/pdb6SKA/pdb], in orange)[1,2] and latrophilin-FLRT (ankyrin-repeat domain, PDB:5FTU [https://doi.org/10.2210/pdb5FTU/pdb], in magenta)[3] binding sites for each splicing-dependent compact dimer. In all panels, coloured ellipses indicate the type of homophilic compact dimeric contacts formed, according to the interface colour codes in Fig. 5E. The rigid-body models of each of the EGF-Ig stalks were calculated using the SAXS data of each isoform (Fig. 5 and Supplementary Fig. 8E). Transmembrane domains are indicated by tubes. The crosses indicate the unfavourable orientation of latrophilin in the teneurin A_0B_1-latrophilin *trans* model.

Supplementary References

1. del Toro D, Carrasquero-Ordaz MA, Chu A, et al. Structural Basis of Teneurin-Latrophilin Interaction in Repulsive Guidance of Migrating Neurons. *Cell*. 2020;180(2):323-339.e19. doi:10.1016/j.cell.2019.12.014

2. Li J, Xie Y, Cornelius S, et al. Alternative splicing controls teneurin-latrophilin interaction and synapse specificity by a shape-shifting mechanism. *Nat Commun*. 2020;11(1). doi:10.1038/s41467-020-16029-7

3. Jackson VA, Mehmood S, Chavent M, et al. Super-complexes of adhesion GPCRs and neural guidance receptors. *Nat Commun*. 2016;11184(7):1-13. doi:10.1038/ncomms11184
