# Supplementary material for: Alternative splicing controls teneurin-3 compact dimer formation for neuronal recognition
Source: Nat Commun. 2024 Apr 29;15:3648. doi: 10.1038/s41467-024-47763-x (PMC11058771; doi:10.1038/s41467-024-47763-x)
Supplement: Supplementary file 3 — Description of Additional Supplementary Files [file 41467_2024_47763_MOESM3_ESM.pdf]

<u>Description of Additional Supplementary Files</u>

File Name: Supplementary Data 1

Description: Table 1: Cryo-EM data collection and refinement statistics.

File Name: Supplementary Data 2

Description: Table2: SAXS data collection parameters and analysis.

File Name: Supplementary Movie 1

Description: Zeroth component of 3D variability analysis of teneurin-3 A1B1 compact dimer performed on the particle set comprising particles both open and closed conformations.

File Name: Supplementary Movie 2

Description: First component of 3D variability analysis of teneurin-3 A1B1 compact dimer performed on the particle set comprising particles both open and closed conformations.
