# Supplementary material for: Alternative splicing controls teneurin-3 compact dimer formation for neuronal recognition
Source: Nat Commun. 2024 Apr 29;15:3648. doi: 10.1038/s41467-024-47763-x (PMC11058771; doi:10.1038/s41467-024-47763-x)
Supplement: Supplementary file 8 — Reporting summary [file 41467_2024_47763_MOESM8_ESM.pdf]

nature portfolio

Corresponding author(s): Dr. Dimphna H. Meijer

Last updated by author(s): Mar 26, 2024

Reporting Summary

Nature Portfolio wishes to improve the reproducibility of the work that we publish. This form provides structure for consistency and transparency in reporting. For further information on Nature Portfolio policies, see our Editorial Policies and the Editorial Policy Checklist.

Statistics

For all statistical analyses, confirm that the following items are present in the figure legend, table legend, main text, or Methods section.

n/a	Confirmed	
☐	☒	The exact sample size (*n*) for each experimental group/condition, given as a discrete number and unit of measurement
☐	☒	A statement on whether measurements were taken from distinct samples or whether the same sample was measured repeatedly
☐	☒	The statistical test(s) used AND whether they are one- or two-sided<br>*Only common tests should be described solely by name; describe more complex techniques in the Methods section.*
☒	☐	A description of all covariates tested
☒	☐	A description of any assumptions or corrections, such as tests of normality and adjustment for multiple comparisons
☐	☒	A full description of the statistical parameters including central tendency (e.g. means) or other basic estimates (e.g. regression coefficient) AND variation (e.g. standard deviation) or associated estimates of uncertainty (e.g. confidence intervals)
☐	☒	For null hypothesis testing, the test statistic (e.g. *F*, *t*, *r*) with confidence intervals, effect sizes, degrees of freedom and *P* value noted<br>*Give P values as exact values whenever suitable.*
☒	☐	For Bayesian analysis, information on the choice of priors and Markov chain Monte Carlo settings
☒	☐	For hierarchical and complex designs, identification of the appropriate level for tests and full reporting of outcomes
☒	☐	Estimates of effect sizes (e.g. Cohen's *d*, Pearson's *r*), indicating how they were calculated

Our web collection on statistics for biologists contains articles on many of the points above.

Software and code

Policy information about availability of computer code

Data collection	EPU
Data analysis	ASTRA 7.3.1<br>RELION 3.1<br>MotionCorr2<br>Gctf 1.06<br>WinCoot 0.9.8<br>Phenix 1.20<br>Chimera 1.15<br>PyMOL 4.6<br>PISA 1.52<br>Molprobity 4.5<br>ATSAS 3.1.3<br>ImageJ 1.54 (Scripts and code used for K562 clustering analysis used in this study are available through Zenodo [https://doi.org/10.5281/zenodo.10843181].)<br>GraphPad Prism 10

For manuscripts utilizing custom algorithms or software that are central to the research but not yet described in published literature, software must be made available to editors and reviewers. We strongly encourage code deposition in a community repository (e.g. GitHub). See the Nature Portfolio guidelines for submitting code & software for further information.

Data

Policy information about availability of data

All manuscripts must include a data availability statement. This statement should provide the following information, where applicable:

- Accession codes, unique identifiers, or web links for publicly available datasets
- A description of any restrictions on data availability
- For clinical datasets or third party data, please ensure that the statement adheres to our policy

The structure model data generated in this study have been deposited in the Protein Data Bank (PDB) under the accession codes 8R50 [https://doi.org/10.2210/pdb8R50/pdb], 8R51 [https://doi.org/10.2210/pdb8R51/pdb], and 8R54 [https://doi.org/10.2210/pdb8R54/pdb]. Previously published structure model data used in this study can be found in the PDB under the accession codes 6FAY [https://doi.org/10.2210/pdb6FAY/pdb], 7BAM [https://doi.org/10.2210/pdb7BAM/pdb], 6SKA [https://doi.org/10.2210/pdb6SKA/pdb], and 5FTU [https://doi.org/10.2210/pdb5FTU/pdb]. The cryo-EM density map data generated in this study have been deposited in the Electron Microscopy Data Bank (EMDB) under the accession codes EMD-18889 [https://www.ebi.ac.uk/emdb/EMD-18889], EMD-18890 [https://www.ebi.ac.uk/emdb/EMD-18890] EMD-18891 [https://www.ebi.ac.uk/emdb/EMD-18891], EMD-18900 [https://www.ebi.ac.uk/emdb/EMD-18900], EMD-18902 [https://www.ebi.ac.uk/emdb/EMD-18902], EMD-19409 [https://www.ebi.ac.uk/emdb/EMD-19409]. See Table 1 for specifications. The SAXS data generated in this study have been deposited in the Small Angle Scattering Biological Data Bank (SASBDB) under the accession codes SASDTY2 [https://www.sasbdb.org/data/SASDTY2/], SASDTZ2 [https://www.sasbdb.org/data/SASDTZ2/], SASDT23 [https://www.sasbdb.org/data/SASDT23/], SASDT33 [https://www.sasbdb.org/data/SASDT33/], SASDT43 [https://www.sasbdb.org/data/SASDT43/], SASDT53 [https://www.sasbdb.org/data/SASDT53/], SASDT63 [https://www.sasbdb.org/data/SASDT63/], SASDT73 [https://www.sasbdb.org/data/SASDT73/], SASDT83 [https://www.sasbdb.org/data/SASDT83/], SASDT93 [https://www.sasbdb.org/data/SASDT93/], SASDTA3 [https://www.sasbdb.org/data/SASDTA3/], SASDTB3 [https://www.sasbdb.org/data/SASDTB3/], SASDTC3 [https://www.sasbdb.org/data/SASDTC3/], SASDTD3 (https://www.sasbdb.org/data/SASDTD3/), SASDTE3 [https://www.sasbdb.org/data/SASDTE3/], SASDTF3 [https://www.sasbdb.org/data/SASDTF3/], SASDTG3 [https://www.sasbdb.org/data/SASDTG3/], SASDTH3 [https://www.sasbdb.org/data/SASDTH3/], SASDTJ3 [https://www.sasbdb.org/data/SASDTJ3/]. See Table 2 for specifications. Source data are provided with this paper.

Research involving human participants, their data, or biological material

Policy information about studies with human participants or human data. See also policy information about sex, gender (identity/presentation), and sexual orientation and race, ethnicity and racism.

Reporting on sex and gender	n.a.
Reporting on race, ethnicity, or other socially relevant groupings	n.a
Population characteristics	n.a.
Recruitment	n.a.
Ethics oversight	n.a.

Note that full information on the approval of the study protocol must also be provided in the manuscript.

Field-specific reporting

Please select the one below that is the best fit for your research. If you are not sure, read the appropriate sections before making your selection.

☒ Life sciences ☐ Behavioural & social sciences ☐ Ecological, evolutionary & environmental sciences

For a reference copy of the document with all sections, see nature.com/documents/nr-reporting-summary-flat.pdf

Life sciences study design

All studies must disclose on these points even when the disclosure is negative.

Sample size	Sample sizes were based on previous experience and conventions in the relevant fields of biophysics and cell biology.
Data exclusions	SAXS protein dilution conditions for which insufficient coherent frames were collected to reliably calculate an averaged scattering curve were excluded, with a maximum of one exclusion per protein sample.
Replication	Number of replications are stated with each experiment in the manuscript. Independent experiments were repeated at least three times to confirm reproducibility.
Randomization	Data for stripe assays and K562 clustering assays were manually randomized. No randomization was applied to other experiments.
Blinding	Data for stripe assays and K562 clustering assays were blindly scored and analyzed, respectively. No blinding of analysis or acquisition was applied to other experiments.

Reporting for specific materials, systems and methods

We require information from authors about some types of materials, experimental systems and methods used in many studies. Here, indicate whether each material, system or method listed is relevant to your study. If you are not sure if a list item applies to your research, read the appropriate section before selecting a response.

Materials & experimental systems

n/a	Involved in the study
☐	☒ Antibodies
☐	☒ Eukaryotic cell lines
☒	☐ Palaeontology and archaeology
☐	☒ Animals and other organisms
☒	☐ Clinical data
☒	☐ Dual use research of concern
☒	☐ Plants

Methods

n/a	Involved in the study
☒	☐ ChIP-seq
☒	☐ Flow cytometry
☒	☐ MRI-based neuroimaging

Antibodies

Antibodies used	1. Alkaline Phosphatase (AP)-conjugated anti-DIG antibody (Neuron visualisation, polyclonal, Isotype: IgG, 1:2000, Roche, 11093274910)<br>2. Anti-6xHis Tag antibody (teneurin-3-his immobilisation stripe assay, 10 ug/ml, monoclonal, Clone: HIS17 GeneTex, GTX44514)<br>3. Goat anti-rabbit AlexaFluor 568 secondary antibody (Stripe visualisation, polyclonal, Isotype: IgG, 10 ug/ml, ThermoFisher, A-11011)
Validation	1. Validation Alkaline Phosphatase (AP)-conjugated anti-DIG antibody (company): "After immunization with digoxigenin, the sheep IgG was purified by ion-exchange chromatography and the specific IgG was isolated by immunosorption. The Fab fragments obtained by papain digestion were conjugated with alkaline phosphatase (AP) and stabilized in 50 mM triethanolamine buffer, 3 mM NaCl, 1 mM"<br>2. Validation goat anti-rabbit AlexaFluor 568 secondary antibody (company): "Anti-Rabbit secondary antibodies are affinity-purified antibodies with well-characterized specificity for rabbit immunoglobulins"<br>3. Validation anti-6xHis Tag antibody (company): "Immunisation done with His6-conjugated to KLH, purification done with Protein-G affinity purification. Validation through western blot analysis."

Eukaryotic cell lines

Policy information about cell lines and Sex and Gender in Research

Cell line source(s)	K562 (DSMZ, 53 year-old female)<br>HEK-E (U-protein Express)
Authentication	We did not perform additional authentication test on the purchased cell lines
Mycoplasma contamination	Cells were tested regularly for mycoplasm contamination, and were tested negative.
Commonly misidentified lines (See ICLAC register)	No commonly misidentified cell lines were used in this study.

Animals and other research organisms

Policy information about studies involving animals; ARRIVE guidelines recommended for reporting animal research, and Sex and Gender in Research

Laboratory animals	Chick embryo's for neuronal explants in stripe assay: Gallus gallus; White Leghorn strain; development day (E) 6; no identification of male or female is possible at embryonic day 6 when the tissue was collected; 3-5 embryos were dissected per condition, done in three independent rounds.
Wild animals	n.a.
Reporting on sex	Both male and female eggs were used indiscriminately. No identification of male or female is possible at embryonic day 6, when the tissue was collected.
Field-collected samples	n.a.
Ethics oversight	All animal work was approved by King's College London Animal Welfare and Ethics Board (AWERB) under the Project licence 70/9036 to RH. However, no ethics protocol is needed for tissue collection at chick embryonic day 6.

Note that full information on the approval of the study protocol must also be provided in the manuscript.

Plants

Seed stocks	n.a.
Novel plant genotypes	n.a.
Authentication	n.a.
